# Metaheuristic Parameter Identification of Motors Using Dynamic Response Relations

**DOI:** 10.3390/s22114050

**Published:** 2022-05-27

**Authors:** Omar Rodríguez-Abreo, Juvenal Rodríguez-Reséndiz, José Manuel Álvarez-Alvarado, Alfonso García-Cerezo

**Affiliations:** 1 Space Robotics Laboratory, Department of Systems Engineering and Automation, Universidad de Malaga, C/Ortiz Ramos s/n, 29071 Malaga, Spain; 2 Faculty of Engineering, Universidad Autónoma de Querétaro, Santiago de Queretaro 76010, Mexico; juvenal@uaq.edu.mx (J.R.-R.); jmalvarez@uaq.mx (J.M.Á.-A.)

**Keywords:** Cuckoo Search, metaheuristic, parameter estimation, DC motor, Jaya, Grey Wolf Optimizer

## Abstract

This article presents the use of the equations of the dynamic response to a step input in metaheuristic algorithm for the parametric estimation of a motor model. The model equations are analyzed, and the relations in steady-state and transient-state are used as delimiters in the search. These relations reduce the number of random parameters in algorithm search and reduce the iterations to find an acceptable result. The tests were implemented in two motors of known parameters to estimate the performance of the modifications in the algorithms. Tests were carried out with three algorithms (Gray Wolf Optimizer, Jaya Algorithm, and Cuckoo Search Algorithm) to prove that the benefits can be extended to various metaheuristics. The search parameters were also varied, and tests were developed with different iterations and populations. The results show an improvement for all the algorithms used, achieving the same error as the original method but with 10 to 50% fewer iterations.

## 1. Introduction

Parameter identification is a highly studied problem due to its multiple fields of application. For example, Hammerstein controlled auto-regressive moving average systems and autonomous robot navigation in the state of charge estimation of lithium-ion batteries [1,2,3]. One of the studied systems for their parametric identification is the direct current motors due to their increasing use in high-demand applications [4].

The parametric identification techniques are diverse. Some authors opt for using intelligent methods, as in [5], where a backpropagation network is used for parametric identification. The disadvantage is that a large amount of data is required to train accurately the network, which is a weakness of all ANNs of this type. Another intelligent technique was used in [6], where the authors estimate the parameters with fuzzy techniques. In [7], a Sparse estimation is developed by combining SPARSEVA and the Steiglitz–McBride method. Methods such as least squares and Steiglitz–McBride are heuristic methods that generally provide adequate results. However, metaheuristic algorithms represent a more straightforward option in complex problems [8]. This optimization algorithm is biologically inspired, and its use is widespread in multiple areas [9].

Metaheuristic algorithms have been widely developed and implemented in multiple areas. These algorithms are iterative which complicates their implementation in real-time [10]. However, its complexity is reduced with respect to other heuristic methods [8]. Some examples of applications are found in works such as [11,12], where authors use metaheuristics algorithms to tune controllers. In [13], a GA with a neural network convolutional is proposed to successfully indicate the likelihood of microscopy images belonging to different classes. In the estimation of parameters, the metaheuristic algorithms have an application such as that shown in [14].

In [15], the authors use a metaheuristic algorithm improved for parameter estimation in permanent magnet synchronous motor. This methodology is similar; however, the dynamic response of the system is not considered. The work contribution is that using existing relationships both in the transient and the steady-state of the motor response. These relations make it possible to reduce an estimation problem of five random variables (model parameters) to use two and calculate the remaining ones. Reducing random variables allows this type of algorithm to improve since the method must comply with these relationships, taken as restrictions. The use of restrictions in the search improves the performance of this type of algorithm [15]. However, no previous work has used the relations of the transient-state and the steady-state as constraints to improve the parameter search process.

The relevance of this work lies in the fact that the engine is a system of second-order differential equations. Therefore, the method can be extended to systems that are modeled in the same way. Second-order systems represent a high percentage of physical systems in engineering systems [16]. Few systems are of one-order, and higher-order systems are usually reduced to second-order for their analysis [16]. Consequently, the method can be adapted by changing the fitness/cost function and the relations between the equations.

The second contribution is improving the three metaheuristic algorithms (the Cuckoo Search, the Gray Wolf Optimizer, and the Jaya algorithm) with dynamic response relations. The tests in the three algorithms are intended to reveal that these relations can be extended to other algorithms of this type and are not only applied in a particular case. Each of the algorithms were tested in their original version and the version proposed in this research.

The standard procedure to develop the parametric estimation was used, where the motor step response is taken as input data. The tests were carried out with two motors of known nominal parameters to compare the error of the improved algorithms. Tests were executed first with simulated signals and later with experimental signals to observe the performance of the algorithms against signals with the typical noise associated with the acquisition of signals.

Finally, the processing time, the accuracy, and the number of necessary iterations of each algorithm are compared. The results indicate a substantial improvement in each algorithm, ranging between 3% and 5% reduction in magnitude error compared to the original algorithm until 50% faster. Additionally, the result indicates that the $CS_{P}$ proposed algorithm is the one that converges in the fewest iterations.

The rest of the work is divided as follows. In Section 2, a literature review is presented to provide an introspective of the proposed work. In Section 3, the motor model and the dynamic response relationships, both in their transient and steady-steady parts. Section 3 described the proposed improvement in each algorithm to include the relations described in part 2. In Section 5, the results at the simulation level and using experimental signals are exhibited. Finally, in Section 6, the conclusions remarking on parameter identification are made.

## 2. Related Work

Parameter estimation has already been studied from a different point of view. For example, the parameters of the motors when controlling in the open and closed-loop have been proposed not only for AC electrical machines [17], but also for DC motors [18,19]. Knowing the parameters of the dynamic model helps to perform multiple control and automation tasks. For example, to improve the machining performance of the toolset when speaking in a mechatronic process [20].

Some authors focus their investigations with heuristic methods, such as the works sent in [21,22]. Another option highly used in parametric estimation is metaheuristic algorithms. They are widely used for their versatility and adaptability to multiple problems [8]. Works such as [23,24,25] have explored the analysis of parametric studies of motors with metaheuristic algorithms.

There are a large number of population-based metaheuristic algorithms that can be adapted to parametric estimation. The most extensive metaheuristic algorithm is the genetic algorithms that can be observed as parametric estimators of motors in works such as [26,27]. However, several algorithms were adapted to this aim; for example, the research [28,29,30] uses the dynamic encoding algorithm for searches (DEAS) for parametric estimation of a motor. In the research [31], a search for harmony (HS) is carried out to obtain the inductance model of an electrical machine. Another example is investigation [32], where a PSO approach is applied to have the parameters of an induction motor. In works [33,34], algorithms such as the whale and the bat are used. Some authors explore the advantages of combining two different metaheuristics algorithms [30]. Finally, some authors such as [29] analyze the online implementation of these algorithms as estimators through parallel processing.

Despite the wide variety of similar works for parameter estimation in motors with metaheuristic algorithms, no work has analyzed the use of dynamic motor relationships as search constraints in both the steady-state and the transient-state. This work aims to analyze the effect of its use in the search with different algorithms, which must be fulfilled. In the Table 1 is shown a comparison between several similar works and the present investigation.

The table above shows that the research presented here differs from the related works that use dynamic relationships to search for the parameters. Therefore, this approach can be implemented as an additional improvement in the works mentioned and can be extended to any stable open-loop system.

## 3. Background

This section briefly explains the D.C. motor equations and their dynamic relations, and describes the three metaheuristic algorithms used in this work. The variables involved in the mathematical model of the DC motor are described in Table 2.

### 3.1. DC Motor and Its Dynamic Response

The electric motor is a hybrid system composed of an electrical part and a mechanical part. Its dynamic behavior is observed through the electrical and mechanical equations in Equation (1).

(1) $$\left.\begin{array}{c} v\left(t\right)=Ri\left(t\right)+L\frac{di(t)}{dt}+E\left(t\right)\\ \tau \left(t\right)=J\frac{d\omega (t)}{dt}+B\omega \left(t\right)+T_{L} \end{array}\right\}$$

The set of equations above is related to the two connection equations depicted in Equation (2).

(2) $$\left.\begin{array}{c} E\left(t\right)=K_{e}\omega \left(t\right)\\ \tau \left(t\right)=K_{m}i\left(t\right) \end{array}\right\}$$

**Figure 1:**
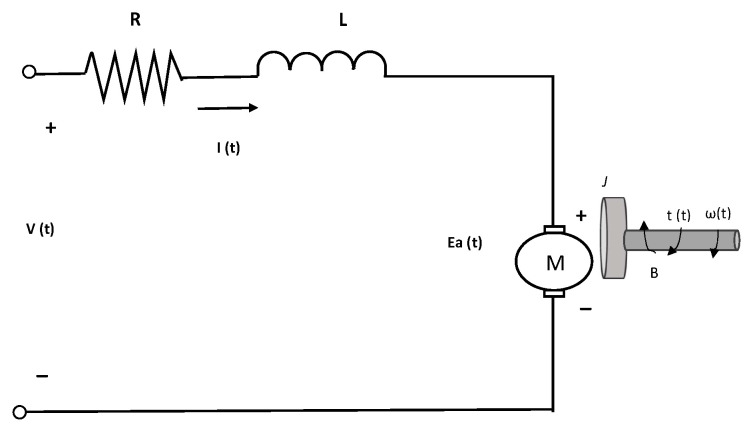
Schematic diagram for a DC motor.

Substituting Equation (2) in Equation (1) and considering a motor without load (*T_L_* = 0), Equation (3) is obtained, and its representation is observed in Figure 1.

(3) $$\left.\begin{array}{c} v\left(t\right)=Ri\left(t\right)+L\frac{di(t)}{dt}+K_{e}\omega \left(t\right)\\ k_{m}i\left(t\right)=J\frac{d\omega (t)}{dt}+B\omega \left(t\right) \end{array}\right\}$$

**Figure 2:**
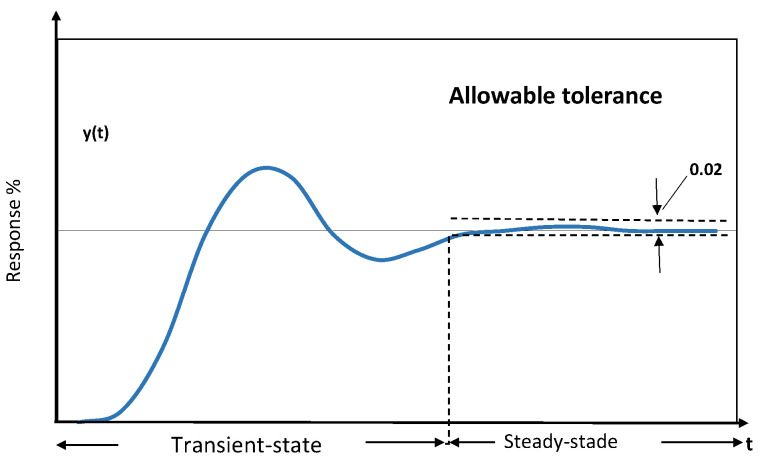
States in dynamic step response.

The previous differential equation system is the model of a DC motor with six unknown parameters. In addition, if null initial conditions are considered, the system can be rewritten in a second-order transfer function. The second-order transfer functions have a well-known typical dynamic step response, as displayed in Figure 2. The dynamic response is divided into two phases, the transient and the steady-state stage.

#### 3.1.1. Steady-State Equations of the Motor

The steady-state is when the output of the system has stabilized, and its response varies less than 2% with respect to time. In this sense, the current and the speed remain constant. Therefore, the derivative of current and the derivative of velocity becomes zero. A fact well-known is that the magnitude of both electrical and mechanical constants is similar. Therefore the same *K* value is used for both. Using these considerations, Equation (3) can be rewritten as:

(4) $$\left.\begin{array}{c} v_{ss}=Ri_{ss}+K\omega_{ss}\\ Ki_{ss}=B\omega_{ss} \end{array}\right\}$$

Equation (4) represents the equations of the motor in steady-state of the six initial parameters, the only parameter that remains in both equations is *K*. Therefore, the system is rewritten, leaving the value of the other parameters as a function of *K*:

(5) $$\left.\begin{array}{c} R=\frac{v_{ss}-K\omega_{ss}}{i_{ss}}\\ B=\frac{Ki_{ss}}{\omega_{ss}} \end{array}\right\}$$

#### 3.1.2. Transient-State Equations of the Motor

The transient part is when the output of the system has variations with respect to time. However, the relationships in this phase are not obvious if it is known that the current has a directly proportional influence on the derivative of the speed. This relation causes the maximum value of the current to cause the maximum value in the derivative of the current, that is:

(6) $$Kmax\left(i\right)=Jmax\left(\frac{d\omega }{dt}\right)+B\omega \left(t_{max(i)}\right)$$

From Equation (6), it can be seen that the parameter *J* can be a function of *B* and *K*, since *B* is already defined as a function of *K*, if *J* is cleared, it is only defined as a function of *K*:

(7) $$J=\frac{B\omega \left(t_{max(i)}\right)-Kmax\left(i\right)}{max(\frac{d\omega }{dt})}$$

Equations (5) and (7) are the relationships of the dynamic response.

### 3.2. Metaheuristics Algorithms

The use of metaheuristic algorithms has spread in the past few years. This is mainly due to the ease of implementation and adaptation to various problems [8]. This work decided to verify the effect of using dynamic relationships in populated-based metaheuristic algorithms. Although multiple tasks can be solved with heuristic methods, the similarity and adaptability to numerous problems and restrictions make metaheuristic algorithms ideal for this study. This work compares three algorithms to determine the effect of dynamic response relations on them.

There are a large number of population-based metaheuristic algorithms in the literature. However, the Cuckoo Search algorithm, the Gray Wolf Optimizer, and the Jaya algorithm were chosen because the first contains an explicit, specific parameter, the GWO has an implicit specific parameter. The Jaya algorithm does not have any specific parameters. Algorithms with two or more specific parameters were avoided because it is required to adjust them. Nevertheless, it should be considered that recent adjustment techniques can solve the problem, such as the hyperparameters adjustment technique, as shown in [35]. The tuning of hyperparameters is another way of improvement for metaheuristics. However, to see only the improvement of the algorithms with the use of dynamic relationships, they were not considered in this work.

#### 3.2.1. Grey Wolf Optimizer

**Figure 3:**
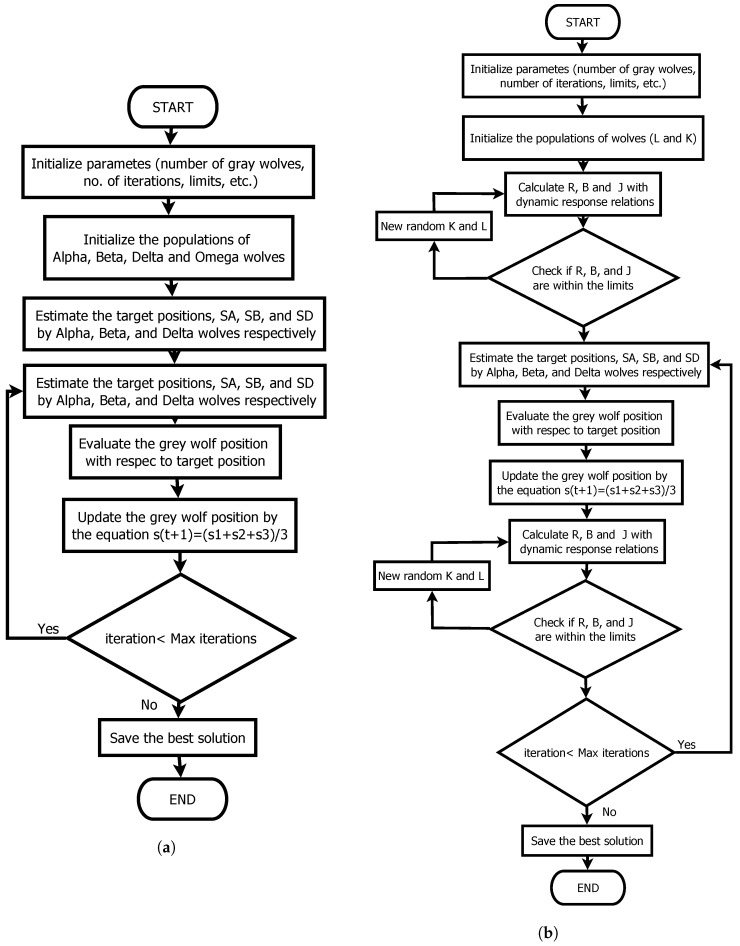
Grey Wolf Optimizer. (**a**) Original Algorithm; (**b**) Modified algorithm with dynamics relations.

The $GWO_{O}$ is inspired by how the wolf hunts its prey. The herd is organized by leading wolves (α) that coordinate the hunt in the space and subordinate wolves (β,δ and $\omega_{wolf}$). The $GWO_{O}$ is fully described in [36], and its process is displayed in Figure 3a. On the other hand, Figure 3b has displayed the $GWO_{P}$ with the dynamic response relations.

The $GWO_{O}$ was chosen among the metaheuristic algorithms since it has a similar performance to the most well-known algorithms in the area, such as Genetic algorithms. It has a simple structure, which allows for fewer computational requirements. It also reduces its search space increasing its convergence speed compared to other algorithms [37].

#### 3.2.2. Algorithm of Jaya

**Figure 4:**
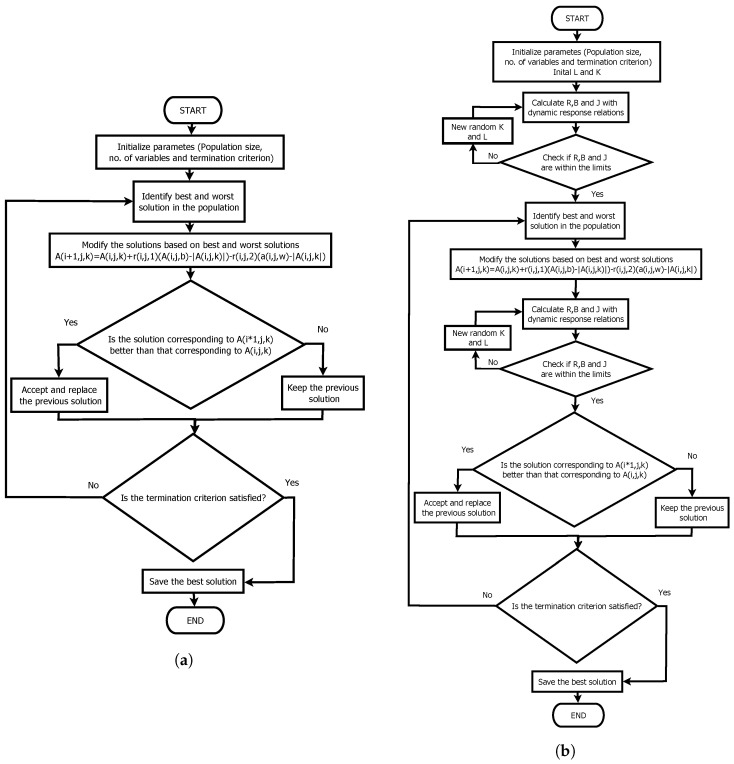
Jaya algorithm. (**a**) Original Algorithm; (**b**) Modified algorithm with dynamics relations.

Jaya is a relatively new algorithm. However, like $GWO_{O}$, it has similar results to the more popular search methods like GA. Its main feature is simplicity since it has only general parameters and no specific parameters. It is only based on the best and worst solution, looking for values close to the best solution and moving away from the worst [38]. The complete method can be studied in detail in [38]. Figure 4a displays the $Jaya_{O}$ while in Figure 4b, the $Jaya_{P}$ with the dynamic relationships of the motor is depicted.

The Jaya algorithm was chosen for reasons similar to the $GWO_{O}$. The algorithm exhibits a fast convergence rate and is among the simplest metaheuristic algorithms to implement.

### 3.3. The Cuckoo Search Algorithm

**Figure 5:**
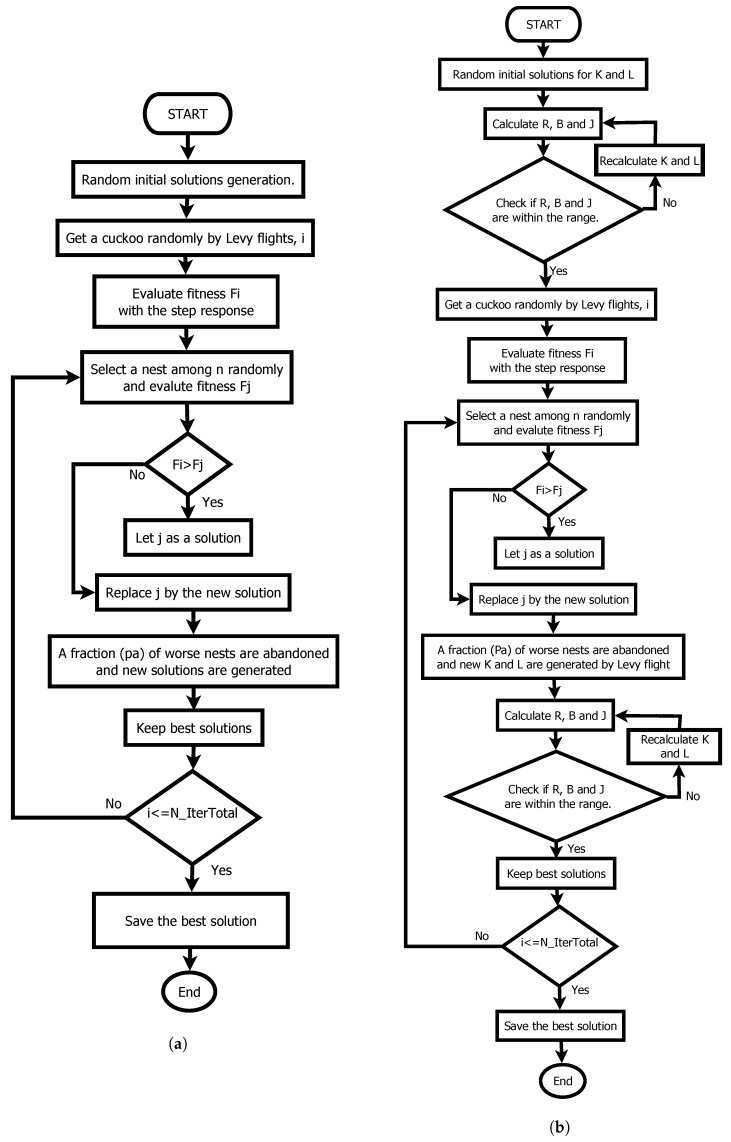
Cuckoo Search algorithm. (**a**) Original Cuckoo search; (**b**) Modified algorithm with dynamics relations.

The $CS_{O}$ is not found among the simplest algorithms unlike the other two algorithms Unlike the other two algorithms. However, like the previous ones, it has shown similar results to the GA, but the convergence is observed in a smaller number of iterations. Like $GWO_{O}$, it is a bioinspired algorithm based on the peculiar way Cuckoo birds reproduce by pinning their eggs in other nests for different birds to raise. Unlike the other two algorithms, the $CS_{O}$ has a specific parameter. Determining the value of any particular parameter is a complex subject of study. However, in the literature, a value of 25% is recommended [39]. The complete method is described in [39] where the author of the algorithm tests the algorithm in problems of a different nature. The differences between the $CS_{O}$ and the $CS_{P}$ can be observed in the flow diagram of both procedures in Figure 5.

The methods used were the Cuckoo Search, the Gray Wolf Optimizer, and the Jaya algorithm was chosen to contrast with the other two algorithms as it has a specific parameter and uses the levy flight hence implementation complexity is greater. However, its performance and application have been tested in multiple areas. Therefore, it is presented as a logical option since, in contrast, the most used metaheuristic (GA) has multiple specific parameters, making a comparison complicated since they must be justified in selecting each parameter.

## 4. Methodology

The methods used were the Cuckoo Search, the Gray Wolf Optimizer, and the Jaya algorithm. All techniques started from original random populations and generated optimal solutions for a fitness/cost function.

The proposal of the present investigation lies in the use of Equation (5) and (7) to reduce the search for random parameters. Instead of searching five parameters simultaneously, only *K* and *L* are randomly generated. For this, the dynamic relations are implemented as search restrictions. In this way, the parameters *J*, *B*, and *R* can be determined by the dynamic equations and as a function of the value proposed by *K* and *L*. Thanks to this, the algorithm focuses only on finding these last two parameters.

In this work, the fitness/cost function is the Euclidean distance between the sum of error in current and the sum of error in velocity, that is:

(8) $$f=\sqrt{\sum {\left(i_{r}-i_{s}\right)}^{2}+\sum {\left(w_{r}-w_{s}\right)}^{2}}$$

The goal of metaheuristic algorithms is to reduce the value of the Equation (8). The three methods are population-based and have several candidate solutions that report data about the search, causing abrupt jumps in the direction of the hopeful solution. In addition, having an initial population with a high number of individuals generates a greater diversity which helps to avoid a local optimum [40].

All metaheuristic algorithms contain parameters that modify their performance. Some parameters are similar or even equivalent. On the other hand, some parameters are specific for each type of method. Table 3 shows the parameters used for each algorithm with the original names given by the respective author and the similar or equivalent parameters.

The three algorithms have the distinction of having a reduced number of parameters, and most of these have their equivalence in other algorithms (see Table 3). This characteristic is one of the reasons for which they were chosen.

The tests of the original and modified algorithms with the dynamic relationships are developed (a total of six algorithms). In addition, two different DC motors will be used to analyze the performance. As a performance measure of each algorithm, the value of the cost function that it reaches was taken, with a cost of 0 being the maximum possible performance. Another factor measured in the tests is the time it takes for the algorithm to complete the search task. The population parameters and maximum iterations were varied by performing the following tests:

- Test 1: Population of 50 individuals and 100 iterations.
- Test 2: Population of 30 individuals and 100 iterations.
- Test 3: Population of 50 individuals and 50 iterations.
- Test 4: Population of 30 individuals and 50 iterations.

A large number of individuals provides greater diversity, and a large number of iterations provides an exhaustive search. However, it should be considered that a greater number of individuals and iterations increasing the computational cost exponentially. The chosen values show allow observing the change in results between runs with twice the number of individuals or iterations. The other common parameters were remain fixed for all the tests. As shown in Table 3, the cost function was in common for the six algorithms, which is determined by Equation (8). The number of variables to be proposed randomly is two (K and L), and the number of variables to be calculated is three (R, B, and J). The initial search limits will also be the same for all algorithms. The upper limit is determined by Ub = [5 0.1 0.5 0.001 0.001] and the lower limit for Lb = [0.1 0.01 0.005 0.0000001 0.00001] corresponding to the vector [R, K, L, J, B].

The first motor used is the Robokits RMCS2004 motor with the following nominal parameters = [0.921042 Ω, 0.073472, 0.007759 H, 0.000136 Nm, 0.000678 $\frac{{\mathrm{Kgm}}^{3}}{s^{2}}$], referred to in this work as M1. Motor 2 is the Mavilor CLM-050 (M2) with the following nominal parameters = [3.1363 Ω, 0.048774, 0.01307 H, 0.000009 Nm, 0.000169 $\frac{{\mathrm{Kgm}}^{3}}{s^{2}}$], this motor is referred in this work as M2.

The tests are are performed with step response signals obtained with simulation and measured by sensors and a data acquisition system. The signals obtained by simulation are called simulated signals and those obtained by measurements are called experimental signals. For the simulation tests, the motor model described by Equation (3) is simulated by Simulink, while the metaheuristic algorithms are developed using Matlab. Thus Matlab obtains a random combination of each method and sends the data to Simulink, which returns a current and velocity signal to Matlab. The software calculates the dynamic relationships and estimates a cost according to each algorithm with these signals. In the case of the experimental signals, only measured data were used.

## 5. Results

In this section, the results obtained for both tests carried out with simulated signals, and those with experimental signals. The following results were found:

- the use of dynamic relationships and the estimation of parameters through metaheuristic algorithms improve the search speed of the parameters;
- this limits the number of results to valid combinations for all parameters.

### 5.1. Results with Simulation Signals

The results of the tests performed with original and proposed algorithms are seen in Table 4.

**Figure 6:**
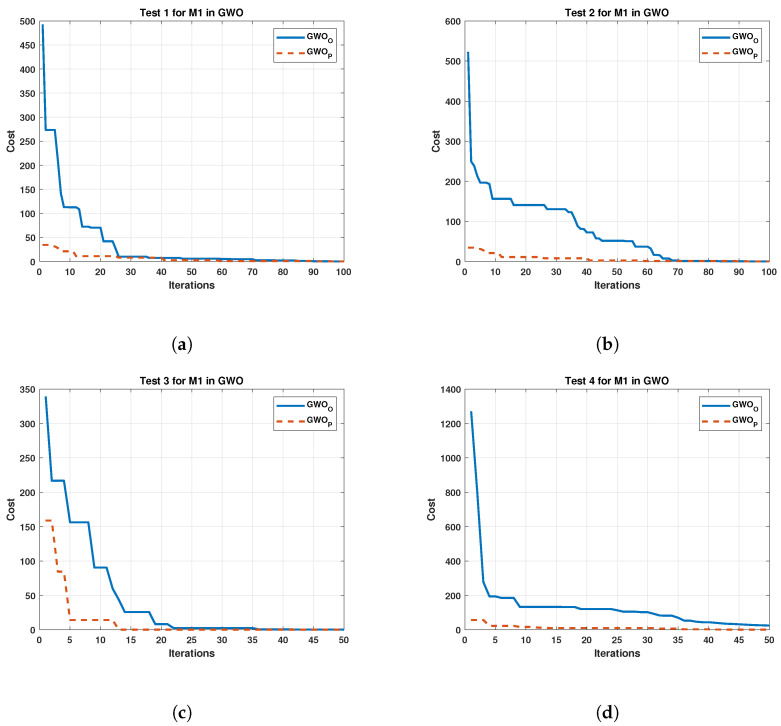
Comparison between the Original and the Gray Wolf Optimizer Proposed in four test for Motor 1. (**a**) cost and iterations of the GWO in test 1 for Motor 1; (**b**) cost and iterations of the GWO in test 2 for Motor 1; (**c**) cost and iterations of the GWO in test 3 for Motor 1; (**d**) cost and iterations of the GWO in test 4 for Motor 1.

The performance of each algorithm in the different tests was represented in the following graphs. Figure 6 displays the performance of $GWO_{O}$ against the $GWO_{P}$ for the M1.

**Figure 7:**
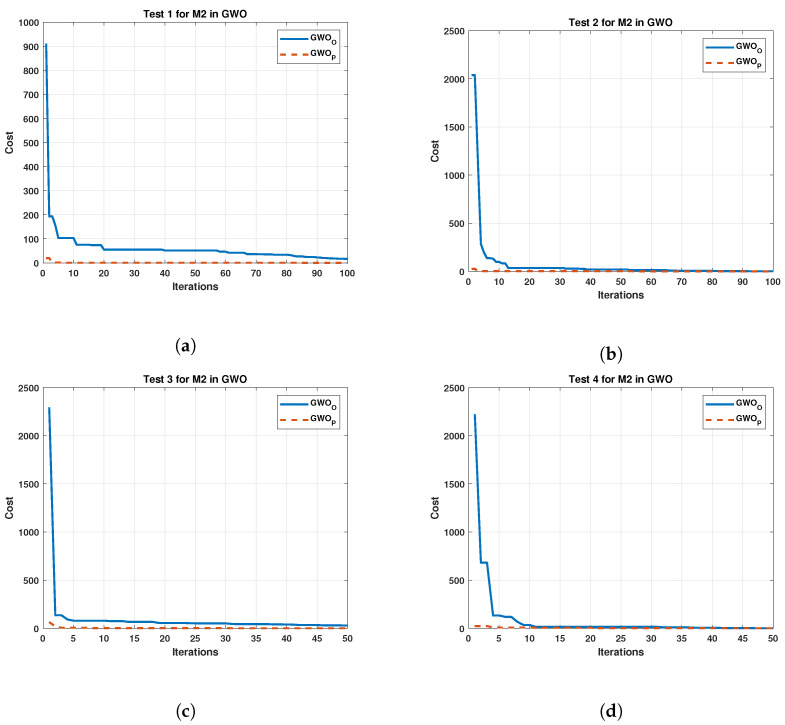
Comparison between the Original and Proposed Gray Wolf Optimizer in four test for Motor 2. (**a**) cost and iterations of the GWO in test 1 for Motor 2; (**b**) cost and iterations of the GWO in test 2 for Motor 2; (**c**) cost and iterations of the GWO in test 3 for Motor 2; (**d**) cost and iterations of the GWO in test 4 for Motor 2.

The test results for Motor 2 are exhibit in Figure 7.

**Figure 8:**
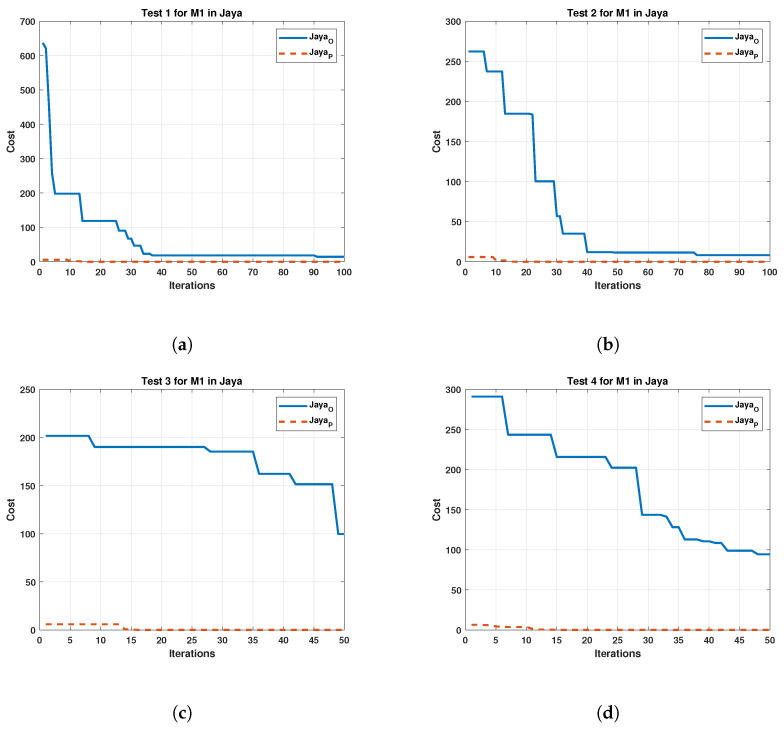
Comparison between the Original and the Proposed Jaya in four test for Motor 1. (**a**) cost and iterations of the Jaya in test 1 for Motor 1; (**b**) cost and iterations of the Jaya in test 2 for Motor 1; (**c**) cost and iterations of the Jaya in test 3 for Motor 1; (**d**) cost and iterations of the Jaya in test 4 for Motor 1.

Both Figure 6 and Figure 7 present an improvement for the $GWO_{O}$, achieving a noticeable initial reduction in cost. The $GWO_{P}$ shows a cost reduction below the original in the following iterations. On the other hand, Figure 8 exhibits the results of the algorithm of Jaya in tests carried out in M1.

**Figure 9:**
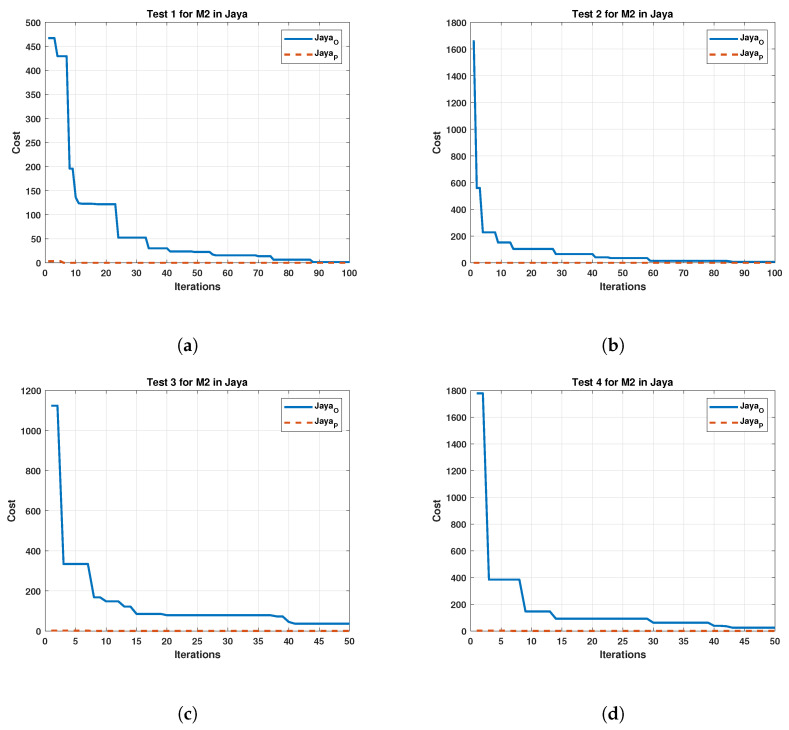
Comparison between the original and the proposed Jaya in four test for Motor 2. (**a**) cost and iterations of the Jaya in test 1 for Motor 2; (**b**) cost and iterations of the Jaya in test 2 for Motor 2; (**c**) cost and iterations of the Jaya in test 3 for Motor 2; (**d**) cost and iterations of the Jaya in test 4 for Motor 2.

The Jaya algorithm results have a similar effect as the GWO, which significantly improves the initial iterations. For tests 1 and 2, the cost reduces similarly in both cases as the iterations increase. However, in cases 3 and 4, the original algorithm does not have proper convergence, unlike the proposed one. Similarly, Figure 9 displays the results obtained by Jaya for M2.

**Figure 10:**
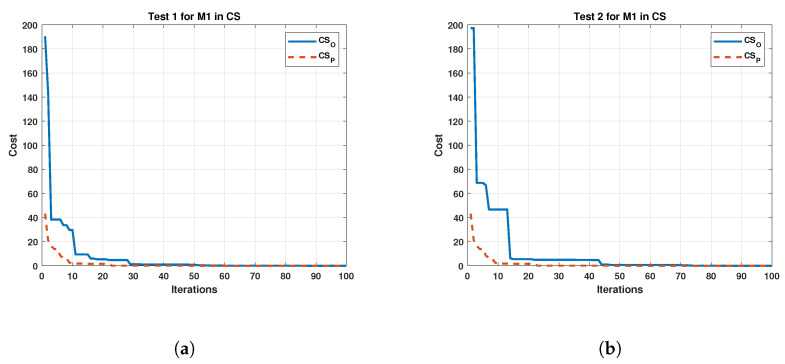

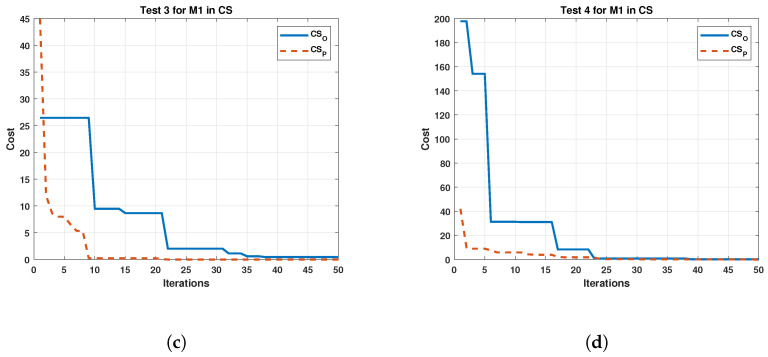
Comparison between the original and the proposed Cuckoo Search in four tests for Motor 1. (**a**) cost and iterations of the Cuckoo Search in test 1 for Motor 1; (**b**) cost and iterations of the Cuckoo Search in test 2 for Motor 1; (**c**) cost and iterations of the Cuckoo Search in test 3 for Motor 1; (**d**) cost and iterations of the Cuckoo Search in test 4 for Motor 1.

Considering the results of Jaya in motor 2, a similar behavior to motor 1 is observed, suggesting that the motor used does not affect the algorithm. Finally, the results of the Cuckoo search algorithm are shown in Figure 10.

**Figure 11:**
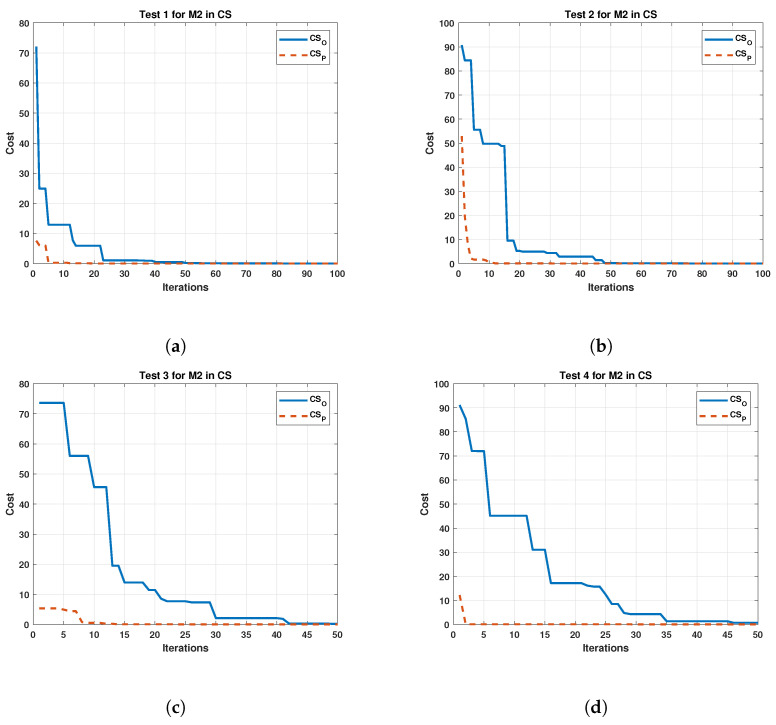
Comparison between the original and the proposed Cuckoo Search in four test for Motor 2. (**a**) cost and iterations of the Cuckoo Search in test 1 for Motor 2; (**b**) cost and iterations of the Cuckoo Search in test 2 for Motor 2; (**c**) cost and iterations of the Cuckoo Search in test 3 for Motor 2; (**d**) cost and iterations of the Cuckoo Search in test 4 for Motor 2.

In the same way, the results of the tests on the M2 are depicted in Figure 11.

The absolute percentage error was calculated in each test with respect to the nominal parameters to observe the performance of the algorithm in terms of precision. The results were summarized in Table 5 and Table 6 for M1 and M2, respectively.

**Figure 12:**
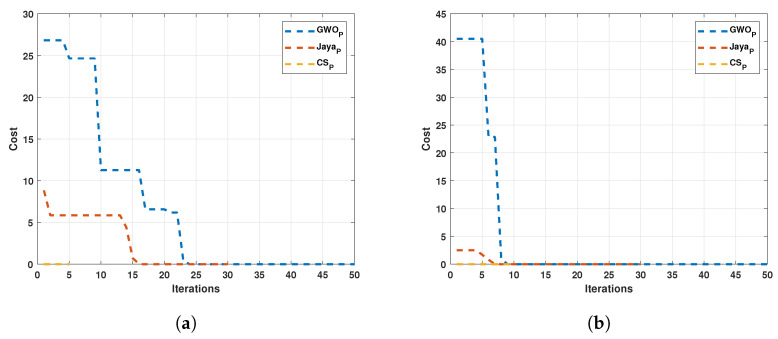
Comparison between the three modified algorithms. (**a**) Tests for Motor 1; (**b**) Tests for Motor 1.

Both numerical and tabular improvements are observed in the modified algorithms compared to the originals. Therefore, subsequent results will focus on the modified algorithms. According to the observed results, the modified algorithm convergence is faster, adjusting the cost curves in the first iterations. Considering the adjustment speed was programmed, a condition for when the cost function obtains a value less than 0.1, the search stopped. The next test was executed in this way for the algorithm convergence speed comparison and was developed with 30 individuals for all algorithms. The results are displayed in Figure 12. Figure 12a shows the convergence speed in iterations for Motor 1, and Figure 12b depicted the convergence speed in iterations for Motor 2.

It can be observed that convergence in the least number of iterations occurs in the Cuckoo search algorithm.

### 5.2. Results with Experimental Signals

**Figure 13:**
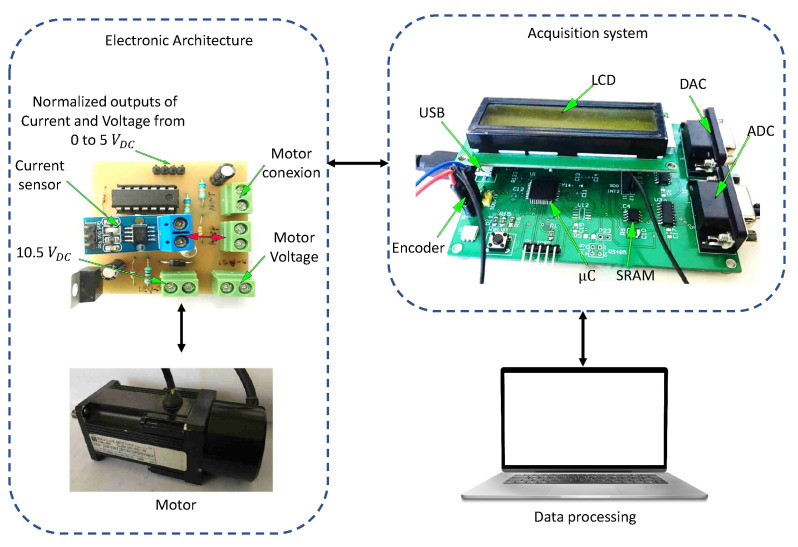
Data acquisition system used for the experimental setup.

In this section were tested the proposed algorithms with experimental signals using an acquisition system of our design based in a simple PIC18F4550 with a sampling period of 0.001 s and implemented in M2. The algorithms work with experimental signals to verify the performance of the three modified algorithms under real conditions, unlike the previous section, the signals acquired from the real world usually have noise and a higher uncertainty which is a function of the measurement system and the conditions in which the measurement was taken. For this case, all algorithms were used with a population of 30 individuals and a maximum of 50 iterations. The sampling process was as follows: at 0.5 s, a voltage of 10.5 constant was applied, and the measured variables were voltage, current, and motor speed, for which a 12-bit ADC, the Hall effect sensor Acs712 and the encoder integrated to the motor are used. Figure 13 displays the setup of the acquisition system.

**Figure 14:**
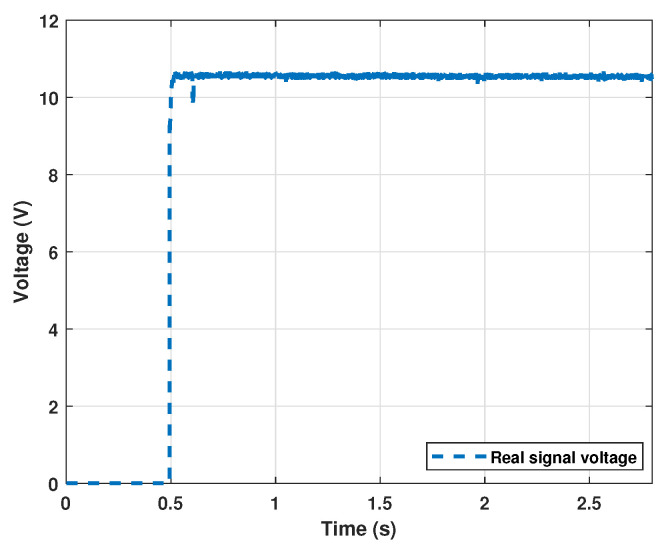
Step voltage signal used.

Although signals can be acquired with such basic equipment, a faster acquisition card and better performing current sensor would help to obtain better quality signals. The results of the acquisition for the step signal used can be observed in Figure 14.

**Figure 15:**
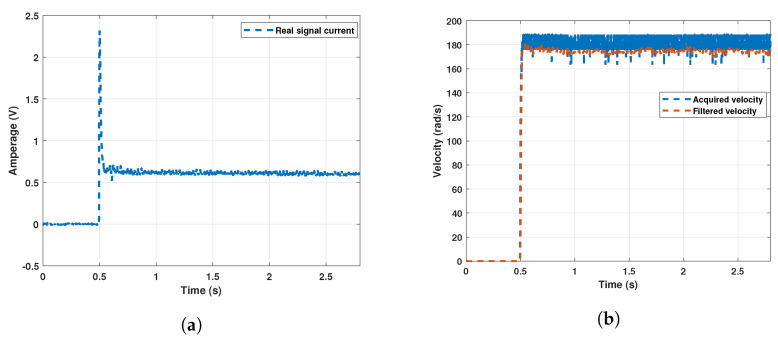
Sensed velocity and current signals for Motor 1. (**a**) Current signal of Motor 1; (**b**) Velocity signal of Motor 1.

Figure 15 shows the current and velocity signals. Due to the acquisition conditions, the speed signal must be filtered. For this, the Chebyshev type 1 digital filter is used. Excessive noise is characteristic of the signal acquisition system and the sensor used. Improving the acquisition hardware would give better signals. However, this is not always possible.

**Figure 16:**
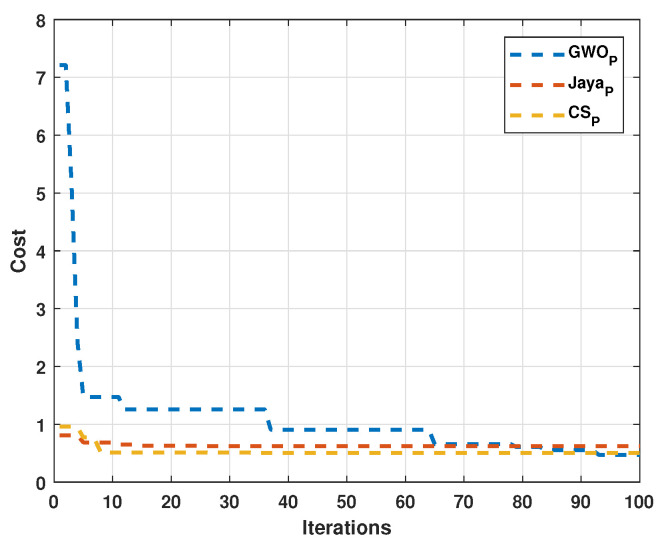
Performance of the proposed algorithms with experimental signals.

With these signals, the dynamic response relations are calculated. For estimating the final current and the final speed, the last 20 values are averaged since the signals preset minor variations, unlike the simulated signals. Again, these variations are attributed to the hardware. The performance of each algorithm can be seen in Figure 16.

**Figure 17:**
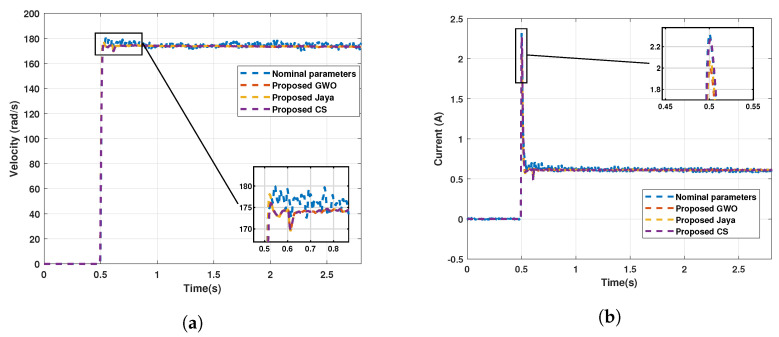
Comparison between the three modified algorithms for experimental signals. (**a**) Comparison between velocities estimates and acquired velocity; (**b**) Comparison between currents estimates and acquired current.

Figure 17 displays the comparison between the current signal and the speed signal calculated for the three modified algorithms with respect to the acquired signal.

The results of experimental signals acquired with noise suggest that the modified algorithms can correctly estimate the parameters to recreate the current and velocity signals.

## 6. Conclusions

This article presents the use of dynamic response relations as search constraints in metaheuristic algorithms used as parametric estimators. The three relationships were used, two in the steady-state and one in the transient-state. It was implemented in three different algorithms with two plants to test the validity of the use of relations. The three relationships founded were used, two in the steady-state and one in the transient state. The proposed method is adaptable to any system with a stable response to the step input, and has at least one relationship in dynamic response. Therefore, this method developed in DC motors can be extended to systems with similar models.

DC motor modeling is a widely studied topic. In particular, the parameter identification with metaheuristic algorithms has shown satisfactory results with multiple techniques. However, the study with the dynamic relationships used as search restrictions has not been studied and most of the improvement techniques are oriented to the optimization of hyperparameters (see Table 1). The relations were implemented in three different algorithms with two plants to test the validity of their implementation.

In general terms, the results and the improvement in each algorithm depend on the initial values that the algorithm takes. Being an algorithm that starts from random values, a precise improvement cannot be quantified. Nevertheless, the results show an evident improvement in each algorithm, especially in the first iterations, where the error is reduced in a smaller number of iterations due to the use of dynamic response relations. Therefore, a lower error and a faster convergence can be observed for all algorithms. The trend holds for different motors, although the performance of the GWO is higher with the M1 than with the M2.

Metaheuristic algorithms with similar or equivalent parameters and with no specific parameters were used to reduce the effect of selecting parameters in different algorithms, achieving a more fair comparison. Reducing iterations and initial errors can help in the implementation in multiple systems since the main problem with metaheuristic algorithms is their high iterative degree, which complicates their implementation.

Metaheuristic algorithms with similar or equivalent parameters and that did not have specific parameters were used to reduce the effect of selecting parameters in different algorithms, achieving a more fair comparison. Reducing iterations and initial errors can help its implementation in multiple systems since the main problem with metaheuristic algorithms is their high iterative degree, which complicates their implementation.

Finally, it should be noted that the metaheuristic algorithms start from random populations, thus the performance can vary according to the initial values that it takes. Four different tests were carried out in two motors to avoid this bias in the three original and modified algorithms. In total, forty-eight tests were developed to verify that cost reduction and iteration reduction are maintained despite the variation of the initial population and the search parameters. Future work should aim to make the tests with the adjustment of hyperparameters and measure execution times with the objective of parallel online implementations.

## Figures and Tables

**Table 1:** Comparison of parameter estimation of DC motor with metaheuristics algorithms in similar works.

Work	Metaheuristic Algorithm	Improvement
Our work	Cuckoo Search Algorithm, Gray Wolf Optimizer, Jaya	Dynamic relations used as search limiter
[15]	Cuckoo Search	Pa adjustment based on fuzzy logic
[23]	Cuckoo Search	Use of steady-state
[26]	Genetic Algorithms	Geared DC motor optimal estimation
[24]	Smart Collaborative Performance	Quantify the contribution of the solutions
[25]	Gray Wolf Optimizer	Torque estimation
[26]	Genetic Algorithms	Optimal estimation
[28]	Dynamic Encoding Algorithm for Searches	Control and fault detection
[33]	Whale Optimization Algorithm	Use of WOA as parameter estimator

**Table 2:** Variables involved in the mathematical model of the DC motor.

Parameter	Description
v(t)	Voltage applied to the motor (V)
τ(t)	Torque generated by the motor (Nm)
i(t)	Current consumed by the motor (A)
ω(t)	Rotor angular speed (rad/s)
$T_{L}$	Load torque (N.m)
*R*	Armature resistance (Ω)
*L*	Armature self-inductance (H)
*K*	The equivalent value of both the electrical and the mechanical constant
*B*	Friction coefficient ($\frac{Kgm^{2}}{s^{2}}$)
*J*	Moment of inertia (Nm)

**Table 3:** Name parameters and equivalence in each algorithm.

Parameter	GWO	Jaya	CS	Description
**Population**	Search Agents_no	pop	nest	Number of initial random solution
**Iterations**	Max_iteration	maxGen	N_IterTotal	
**Limits**	lb, ub	mini, maxi	lb, ub	upper and lower boundaries of the search
**Variables to search**	dim	var	nd	Number of variables to search
**Fitness/cost function**	fobj	objective	fobj	Fitness function used (same for three algorithms)
**Pa**	-	-	Pa	Probability of the alien egg being discovered

**Table 4:** Cost/Fitness result for each test in M1 and M2.

Test	Motor Cost	${\mathrm{GWO}}_{O}$ Cost	${\mathrm{GWO}}_{P}$ Cost	${\mathrm{Jaya}}_{O}$ Cost	${\mathrm{Jaya}}_{P}$ Cost	${\mathrm{CS}}_{O}$ Cost	${\mathrm{CS}}_{P}$ Cost
Test 1	M1	0.19	0.08	14.66	4.98 × 10${}^{-4}$	0.003	3.091 × 10${}^{-5}$
Test 2	M1	0.08	0.17	8.25	4.97 × 10${}^{-4}$	0.004	1.232 × 10${}^{-4}$
Test 3	M1	0.12	0.03	99.66	5.63 × 10${}^{-4}$	0.455	4.837 × 10${}^{-4}$
Test 4	M1	24.33	0.07	94.34	7.67 × 10${}^{-4}$	0.170	1.911 × 10${}^{-3}$
Test 1	M2	16.96	0.05	1.59	2.65 × 10${}^{-3}$	0.001	4.973 × 10${}^{-6}$
Test 2	M2	0.88	0.55	7.34	2.65 × 10${}^{-3}$	0.001	1.692 × 10${}^{-5}$
Test 3	M2	28.28	0.13	36.37	2.70 × 10${}^{-3}$	0.194	4.554 × 10${}^{-4}$
Test 4	M2	0.63	0.10	24.52	2.71 × 10${}^{-3}$	0.671	1.434 × 10${}^{-3}$

**Table 5:** The absolute percentage error for test in Motor 1.

Test	Algorithm	*R* %	*K* %	*L* %	*J* %	*B* %
Test 1	$GWO_{O}$	1.557	0.355	6.721	2.317	0.182
	$GWO_{P}$	1.473	0.173	0.591	0.397	0.173
	$Jaya_{O}$	15.081	3.801	55.180	12.917	0.340
	$Jaya_{P}$	0.012	0.001	0.210	0.225	0.001
	$CS_{O}$	0.252	0.029	0.068	0.290	0.029
	$CS_{P}$	0.008	0.001	0.041	0.027	0.001
Test 2	$GWO_{O}$	0.262	0.161	4.030	0.198	0.077
	$GWO_{P}$	0.653	0.076	4.476	0.300	0.076
	$Jaya_{O}$	5.623	0.396	55.180	20.540	0.899
	$Jaya_{P}$	0.013	0.001	0.194	0.225	0.001
	$CS_{O}$	0.198	0.023	1.084	0.226	0.023
	$CS_{P}$	0.022	0.002	0.069	0.098	0.002
Test 3	$GWO_{O}$	1.445	0.020	3.555	0.508	0.391
	$GWO_{P}$	1.355	0.159	1.391	0.383	0.159
	$Jaya_{O}$	20.539	0.572	55.180	168.091	12.048
	$Jaya_{P}$	0.014	0.002	0.300	0.225	0.002
	$CS_{O}$	3.137	0.376	9.907	3.560	0.376
	$CS_{P}$	0.004	0.001	0.212	0.215	0.001
Test 4	$GWO_{O}$	13.269	2.785	53.598	66.302	0.375
	$GWO_{P}$	1.233	0.141	0.437	0.082	0.141
	$Jaya_{O}$	63.585	21.087	57.780	55.245	35.513
	$Jaya_{P}$	0.151	0.018	0.191	0.241	0.018
	$CS_{O}$	0.369	0.043	5.891	3.551	0.043
	$CS_{P}$	0.241	0.028	0.269	0.251	0.028

**Table 6:** The absolute percentage error for test in Motor 2.

Test	Algorithm	*R* %	*K* %	*L* %	*J* %	*B* %
Test 1	$GWO_{O}$	29.525	4.733	61.008	70.750	5.774
	$GWO_{P}$	0.244	0.054	5.024	0.755	0.054
	$Jaya_{O}$	9.779	2.480	3.253	4.529	5.228
	$Jaya_{P}$	0.087	0.020	0.667	0.829	0.020
	$CS_{O}$	0.203	0.045	0.086	0.264	0.045
	$CS_{P}$	0.025	0.005	0.024	0.023	0.005
Test 2	$GWO_{O}$	5.792	1.448	19.533	6.690	1.554
	$GWO_{P}$	2.773	0.640	18.245	1.454	0.640
	$Jaya_{O}$	9.550	1.637	28.856	17.807	0.466
	$Jaya_{P}$	0.060	0.013	0.703	0.823	0.013
	$CS_{O}$	0.307	0.069	0.632	0.459	0.069
	$CS_{P}$	0.053	0.012	0.025	0.013	0.012
Test 3	$GWO_{O}$	11.298	1.530	67.539	192.484	2.660
	$GWO_{P}$	0.936	0.206	8.692	0.601	0.206
	$Jaya_{O}$	6.186	0.080	65.769	162.739	6.260
	$Jaya_{P}$	0.121	0.027	0.637	0.836	0.027
	$CS_{O}$	0.291	0.065	10.560	5.329	0.065
	$CS_{P}$	0.028	0.006	0.551	0.102	0.006
Test 4	$GWO_{O}$	4.496	0.966	12.593	12.692	0.737
	$GWO_{P}$	2.004	0.436	4.146	0.370	0.436
	$Jaya_{O}$	35.942	14.237	161.400	28.310	6.117
	$Jaya_{P}$	0.000	0.000	0.665	0.809	0.000
	$CS_{O}$	4.296	0.909	13.716	11.149	0.909
	$CS_{P}$	0.113	0.025	0.035	0.556	0.025

## Data Availability

The data presented in this study are available on request from the corresponding author.

## Abbreviations

$CS_{O}$	Original Cuckoo Search Algorithm
$WGO_{O}$	Original Gray Wolf Optimizer
$Jaya_{O}$	Original Jaya Algorithm
$CS_{P}$	Proposed Cuckoo Search Algorithm for motor parameter estimation
$WGO_{P}$	Proposed Gray Wolf Optimize for motor parameter estimation
$Jaya_{P}$	Proposed Jaya Algorithm for motor parameter estimation
GA	Genetic algorithm
DC	Direct current
ess	Steady-stade error
*R*	Armature resistance
*L*	Armature self-inductance
$T_{L}$	Load torque
Km	Mechanical constant
Ke	Electrical constant
*B*	Friction coefficient
*J*	Moment of inertia
*K*	The equivalent value of both the electrical and the mechanical constant.
τ(t)	Motor torque
E(t)	Back electromotive force
ω(t)	Angular velocity of rotor
v(t)	Voltage
i(t)	Current
$v_{ss}$	Voltage in steady-state
$i_{ss}$	Current in steady-state
$\omega_{ss}$	Angular velocity in steady-state
max(i)	Maximum current
$\omega (t_{max(i)})$	Time in which the maximum current occurs
*f*	fitness/cost function
Ub	Upper boundary
Lb	Lower boundary
*i*	Current response to the step of motor
$i_{s}$	Current response to the step of the parameters proposed in a simulation
ω	Angular velocity response to the step of motor
$\omega_{s}$	Angular velocity response to the step of the parameters proposed in a simulation
$max(\frac{d\omega }{dt})$	Maximum angular acceleration
